# Listening Comprehension and Listening Effort in the Primary School Classroom

**DOI:** 10.3389/fpsyg.2018.01193

**Published:** 2018-07-12

**Authors:** Mary Rudner, Viveka Lyberg-Åhlander, Jonas Brännström, Jens Nirme, M. K. Pichora-Fuller, Birgitta Sahlén

**Affiliations:** ^1^Linnaeus Centre HEAD, Department of Behavioural Sciences and Learning, Linköping University, Linköping, Sweden; ^2^Department of Clinical Sciences, Logopedics, Phoniatrics and Audiology, Lund University, Lund, Sweden; ^3^Lund University Cognitive Science (LUCS), Lund University, Lund, Sweden; ^4^Department of Psychology, University of Toronto, Toronto, ON, Canada

**Keywords:** effort, motivation, listening comprehension, classroom, context, multi-talker babble noise, dysphonic voice, cognition

## Abstract

In the primary school classroom, children are exposed to multiple factors that combine to create adverse conditions for listening to and understanding what the teacher is saying. Despite the ubiquity of these conditions, there is little knowledge concerning the way in which various factors combine to influence listening comprehension and the effortfulness of listening. The aim of the present study was to investigate the combined effects of background noise, voice quality, and visual cues on children’s listening comprehension and effort. To achieve this aim, we performed a set of four well-controlled, yet ecologically valid, experiments with 245 eight-year-old participants. Classroom listening conditions were simulated using a digitally animated talker with a dysphonic (hoarse) voice and background babble noise composed of several children talking. Results show that even low levels of babble noise interfere with listening comprehension, and there was some evidence that this effect was reduced by seeing the talker’s face. Dysphonia did not significantly reduce listening comprehension scores, but it was considered unpleasant and made listening seem difficult, probably by reducing motivation to listen. We found some evidence that listening comprehension performance under adverse conditions is positively associated with individual differences in executive function. Overall, these results suggest that multiple factors combine to influence listening comprehension and effort for child listeners in the primary school classroom. The constellation of these room, talker, modality, and listener factors should be taken into account in the planning and design of educational and learning activities.

## Introduction

Challenging listening conditions such as background noise, poor signal quality and an immature language system can interfere with spoken language understanding (Mattys et al., 2012) and generate a sense of listening effort that can be understood, at least partly, in cognitive terms (Rönnberg et al., 2013; Pichora-Fuller et al., 2016). Listening effort has been investigated extensively in adults (Pichora-Fuller et al., 2016), but there are fewer studies in children (for a review see McGarrigle et al., 2014). Children as well as adults experience challenging listening situations and the school classroom is a good example of this (Sahlén et al., 2018). The recommended maximum background noise level in classrooms is 35 decibel (dB) sound pressure level (SPL; ANSI/ASA S12.60-2010, 2010). However, this level is often exceeded, and the signal-to-noise ratio (SNR) in the North American classrooms typically varies between −6 and +6 dB (Crandell and Smaldino, 2000). Similar conditions are likely to apply in other developed countries. Other North American studies have shown that background noise impedes complex conversational interaction and collaborative learning in the classroom (McKellin et al., 2011) and causes children with normal hearing to adopt strategies otherwise observed in individuals with hearing impairment (HI; McKellin et al., 2007).

Although the conversational strategies of children with HI are different from those of their normal hearing peers, children with HI are considered active and competent conversational partners, providing that the conversational context is optimized (Sandgren et al., 2015). In the classroom, however, the conversational context is seldom optimized. In general, background noise not only reduces the intelligibility of speech but also a listener’s ability to recall its content (Rabbitt, 1990; Pichora-Fuller et al., 1995; Baldwin and Ash, 2011; Hygge et al., 2015). Classroom noise may compromise the comprehension of spoken language and its subsequent recall, in turn, compromising learning. Furthermore, teachers may strain their voices, becoming dysphonic (hoarse), in an effort to make themselves heard above the noise. Children are at risk of underachievement when trying to understand a dysphonic voice, especially when the task itself is less challenging or the child’s cognitive capacity is stretched (Lyberg-Åhlander et al., 2015). Moreover, a talker with a dysphonic voice may be even harder to understand than a normal voice when there is background noise (Ishikawa et al., 2017) or the talker may be perceived to be less intelligent and less socially attractive (Eadie et al., 2017).

These external sources of listening effort are further complicated by the individual characteristics of the students. As children’s language and cognition are developing, they have less efficient working memory and executive functions and poorer episodic and semantic memory compared to adults (Gathercole et al., 2004). For some children, linguistic and cognitive immaturity is compounded by sensory and/or linguistic and cognitive impairments. Therefore, all children are more reliant on context than are adults when processing information during comprehension and memory or learning tasks (Craik and Bialystok, 2012), and children with sensory, linguistic and cognitive impairments are even more in need of context (Sahlén et al., 2018). Although supportive context in the form of knowledge concerning the physical, social and cultural setting may be readily available in the familiar home environment, it is often less accessible in the classroom. Indeed, to a large degree, learning is about mastering new contexts. Decontextualized language challenges pupils’ listening comprehension by calling for a range of metalinguistic and metacognitive skills, such as understanding word meaning in different contexts, making inferences at the discourse level, understanding genre-specific requirements, self-regulation and theory of mind (Paul and Norbury, 2012, p407–409). Visual information can provide an important source of context that can be used to compensate when listening conditions are adverse. A literature is emerging to suggest that success factors for children with HI in a language-supporting classroom are structured context, no time limit, and a visible and well-known conversational partner in an optimal sound environment (Sahlén et al., 2018). Thus, optimal performance likely depends on a combination of listener, talker and situational factors.

Speech processing in adults proceeds smoothly and effortlessly providing listening conditions are optimal (Mattys et al., 2012); however, when there is a mismatch between the incoming spoken language signal and the individual listener’s cognitive representations, successful comprehension relies on explicit cognitive processing that may be experienced as being effortful (Rönnberg, 2003; Rönnberg et al., 2013; Rudner et al., 2011, 2012). The explicit processing engendered by a mismatch between the perception of the incoming signal and the signal expected based on representations is likely to lead to the establishment of new or altered representations (Rudner and Holmer, 2016; Holmer et al., 2016), which is tantamount to learning.

Seeing the talker’s face improves spoken language comprehension in noise (Sumby and Pollack, 1954) and should therefore release resources for remembering what was said in noise (Rudner et al., 2016). Nevertheless, the evidence is mixed and the long-term benefits of audio-visual over auditory-only input may depend on the age of the listener, as well as his or her cognitive and sensory abilities (Rudner et al., 2016), and motivation (Pichora-Fuller et al., 2016; Sahlén et al., 2018).

Hitherto, there has been little systematic investigation of the way in which seeing the talker’s face can contribute to the language-supporting classroom (Sahlén et al., 2018). Here we present data from four separate experiments investigating the effect of multi-talker child babble noise on comprehension and recall by 8 year-old children of passages spoken by a digitally animated talker with dysphonia in a well-controlled simulated primary school classroom.

## Materials and Methods

### Participants

A total of 245 eight-year-old children (128 females) took part in four separate experiments. They were recruited from local primary schools in the south of Sweden and were reported to have typical language and cognitive development. Children in the first three studies had normal pure-tone thresholds determined by audiometric testing and defined as being no greater than 25 dB HL at 0.5, 1, 2, 3, 4, and 6 kHz. Participants in the fourth study had normal hearing according to parental report. The caregivers of all participants provided written informed consent and the experiments were approved by the Regional Ethical Review Board under registration number 2014/408.

### Auditory Passage Comprehension

All experiments were based on the passage comprehension module of the Clinical Evaluation of Language Fundamentals (CELF 4; Semel et al., 2003), an internationally well-established language assessment test battery for children (Denman et al., 2017). Passage comprehension involves listening to short narrative texts and answering related questions.

The passages were pre-recorded in a sound-treated booth by a female speech-language pathologist with a standard Swedish dialect who spoke at a normal speech rate of 150–169 words per minute (Haake et al., 2014). To achieve the dysphonic voice applied in some of the experimental conditions, the talker underwent a vocal loading procedure as described in Whitling et al. (2015) prior to recording. The dysphonic voice mimics a voice quality that typically occurs in a noisy teaching situation. Voice recording levels across materials were equalized to the same root mean square using Adobe Audition CS6 (Adobe Systems, San José, CA, United States).

Visual support in the form of a digitally animated virtual talker was provided in all four experiments. The virtual talker was generated by capturing facial and postural movements of the model during audio recording of the passages and then implementing them in a digital character (for details see Nirme et al., 2018).

The CELF 4 was presented over sound-attenuating and circumaural earphones (Sennheiser HDA 200) from a laptop computer. After each passage, the experimenter asked the participant five questions to test implicit and explicit comprehension of the text and scored the answers according to the standard CELF 4 test protocol with open questions in Experiments 1–3. In Experiment 4, a multiple-choice procedure was adopted instead.

### Executive Function and Subjective Ratings of Difficulty

Elithorn’s Mazes (EM; WISC IV Integrated, Wechsler, 2004), which is a test of executive function suitable for children was administered to participants in Experiments 2 and 3. Ratings of the perceived difficulty of CELF 4 passages under different conditions were obtained in the first three experiments.

### Experiment 1: Evaluation of the Digitally Animated Virtual Talker

#### Procedure

The participants (*N* = 95, 49 females) were randomized to one of three groups and performed the CELF 4 in one of three conditions: (1) audio–visual with the digitally animated virtual talker; (2) audio–visual with the video-recorded natural talker, or (3) audio-only with no visual support. In all three conditions, the talker’s voice was dysphonic. In all conditions, there was multi-talker babble noise at +10 dB SNR to simulate a somewhat more favorable SNR condition than the typical −6 to +6 dB SNR conditions found in classrooms (Crandell and Smaldino, 2000). The babble noise was produced by combining the recordings of four children reading separate stories.

#### Results

A one-way analysis of variance (ANOVA) showed a statistically significant effect of condition on the CELF 4 score, *F*(2,92) = 4.15, *p* = 0.019. Bonferroni-adjusted, pair-wise *t*-tests showed that the CELF 4 score was significantly better (*p* = 0.020) with the virtual talker, *M* = 10.9, *SD* = 2.40, than with no visual support, *M* = 9.1, *SD* = 2.65, but there was no statistically significant difference between the virtual and natural audio-visual conditions, *M* = 10.4, *SD* = 2.68. There was no significant difference in the rating of perceived difficulty across conditions.

#### Conclusion

A digitally animated virtual talker is at least as effective as a video-recorded natural talker in providing visual support during auditory passage comprehension in a simulated classroom situation. These findings validate the use of the virtual talker as a way of providing supportive visual cues during a speech understanding test.

### Experiment 2: Effect of Noise on Auditory Passage Comprehension With and Without Visual Support

#### Procedure

The participants (*N* = 56; 35 females) performed the CELF 4 under four conditions within a 2 × 2 factorial design with the order of conditions randomized. The four conditions were: visual support (yes, no) and multi-talker babble noise (yes, no). The babble was generated as in Experiment 1. In all conditions, the talker’s voice was dysphonic.

A mixed model linear regression (conditional *R^2^* = 0.295) showed a statistically significant negative effect of multi-talker babble noise on CELF 4 score, β = −0.168, *t* = −4.108, *p* < 0.001. There was no significant effect of visual support in the quiet background, β = −0.0175, *t* = −0.428, *p* = 0.669, nor was there a significant interaction between visual support and multi-talker babble noise, β = 0.070, *t* = 1.210, *p* = 0.228.

Elithorn’s Mazes score positively predicted CELF 4 score in the quiet condition with no visual support, β = 0.012, *t* = 2.525, *p* = 0.012; however, there was a marginally significant negative interaction effect between EM and the CELF 4 score in the multi-talker babble noise, β = −0.011, *t* = −1.837, *p* = 0.068, indicating that the link between EM and CELF 4 score was weakened in the presence of noise. There was no significant interaction effect between EM and visual support, β = −0.0078, *t* = −1.275, *p* = 0.204.

#### Conclusion

There was no evidence that visual support improved the comprehension of passages read by a dysphonic talker in low-level multi-talker babble noise.

### Experiment 3: Effect of Dysphonic Voice on Auditory Passage Comprehension With and Without Visual Support

#### Procedure

The participants (*N* = 57; 25 females) performed CELF 4 under four conditions in randomized order within a 2 × 2 factorial design. The four conditions were: visual support (yes, no) and dysphonic voice (yes, no). Delayed recall based on the CELF 4 was also tested.

#### Results

There were no significant experimental effects on CELF 4 score or recall. However, EM scores correlated significantly with CELF 4 scores, both in the auditory-only conditions, ρ = 0.45, *p* < 0.001, and in the audiovisual conditions, ρ = 0.37, *p* = 0.004, in both cases collapsed across voice quality. When there was no visual support, the dysphonic voice was rated as significantly more difficult to understand than the non-dysphonic voice, *p* = 0.032, and it was also described as being more unpleasant.

#### Conclusion

Compared to a normal voice, a dysphonic voice is perceived to be more unpleasant and increases perceived comprehension difficulty when there is no visual support, even though the type of voice affected neither listeners’ comprehension nor recall of passages. Good executive function seems to be important for auditory passage comprehension irrespective of visual support.

### Experiment 4: Effect on Auditory Passage Comprehension of Audio–Visual Multi-Talker Babble Noise

#### Procedure

The participants (*N* = 36; 19 females) performed the CELF 4 under four conditions with different materials according to a Latin square design. The conditions were (1) auditory-only presentation with no babble noise; (2) auditory-only presentation with multi-talker babble noise; (3) auditory multi-talker babble noise with congruent visual support; (4) auditory multi-talker babble noise with visual information that was incongruent with the noise. The multi-talker babble noise was generated by combining the recordings of two children reading aloud. Data from one participant who did not consistently watch the screen was excluded.

#### Results

Mixed model linear regression analysis showed no significant difference in CELF 4 scores between conditions.

#### Conclusion

This small-scale study provides no evidence that audio–visual background babble (acoustic noise accompanied by visual information) impairs auditory passage comprehension.

## Summary of Findings

In the simulated classroom situation, even a low level of multi-talker babble noise reduces the comprehension of spoken passages and increases perceived difficulty. However, this effect may be alleviated if the target talker’s face is visible. When the talker’s voice was dysphonic, perceived difficulty increased and perceived pleasantness decreased, although passage comprehension was not affected. We found some evidence that good auditory passage comprehension in a simulated classroom situation is associated with good executive function.

## Perspective

The empirical work reported here demonstrates that the simulated classroom conditions we studied reduce listening comprehension, increase perceived listening difficulty and reduce pleasantness. These findings can be understood by applying the Framework for Understanding Effortful Listening (FUEL, Pichora-Fuller et al., 2016). According to the FUEL, successful listening under challenging conditions requires motivation and effort. In particular, when task demands are high (e.g., excessive background noise), motivation is required to apply the effort required for successful listening.

FUEL applies Kahneman (1973) model of effort and attention to listening by simply extending a general definition of effort to the domain of listening. In general, effort is defined as “*the deliberate allocation of mental resources to overcome obstacles in goal pursuit when carrying out a task,”* with listening effort being specific to tasks involving listening (original italics, p 5S). It is interesting to relate our findings about listening by children in classroom-like conditions to the FUEL because it highlights the potential role of motivation in managing the cognitive resources required for active listening in noisy situations.

The FUEL specifies that input-related demands contributing to listening effort may include source factors relating to voice quality, transmission factors such as noise, listener factors such as sensory and cognitive abilities, and contextual factors such as supportive visual information. Further, following Kahneman (1973), according to the FUEL, allocation of resources is influenced by both intentional attention (e.g., following task instructions) and automatic attention (e.g., response to novel stimuli). In terms of attention-related responses, performance on behavioral tasks and self-report are recognized in FUEL as possible ways to measure changes related to listening effort, including behavioral and self-report measures. The input-related factors of FUEL were taken into account in the empirical work reported here on children who performed a listening comprehension task in various conditions corresponding to those that might challenge listening in classrooms. Our procedures and measures also map readily to components in the FUEL.

The FUEL is based on Kahneman’s classic work on attention and effort and it has much in common with other cognitive models; however, because FUEL relates specifically to listening, it was helpful to us to use it as framework within which to interpret our present findings. We have shown that input-related demands, namely the transmission factor (multi-talker child babble noise, even at levels more favorable than those found in many classrooms) reduces auditory passage comprehension in 8-year-olds who have typical language and cognition development. Further, this input-related effect was accompanied by the attention-related response of greater perceived difficulty. These findings suggest that multi-talker babble noise in the classroom can increase the demand for cognitive capacity during the activity of passage comprehension. Further, we found that the source factor “dysphonic voice,” another type of input-related demand, was perceived as more unpleasant and elicited higher difficulty ratings, suggesting increased effort or allocation of cognitive resources to meet the demands placed on the listener during the activity. The rating of the “dysphonic voice” as being more unpleasant may be related to possible effects of motivation on the listener’s allocation of attentional resources to the activity. There was some evidence that the context factor of providing a visible talking face (which can alter input-related demand within the FUEL) can compensate for the increased demand imposed by multi-talker babble noise. In addition, we found some evidence that better executive function was associated with better auditory passage comprehension, supporting the notion featured in the FUEL and other models of cognition that listeners may allocate available capacity to prioritized activities.

These findings relate to children with typical linguistic and cognitive development. However, it is worth noting that many children with disabilities are being integrated in mainstream schools today. For example, in Sweden, 85% of children with HI are integrated into regular classes with large class sizes. The special services and support needed for their academic achievement are often limited. For example, in such mainstream situations, there may not be adequate visual cues (e.g., in the form of signing support for those children who need it) or turn-taking support (e.g., in the form of table microphones or FM room systems). Identifying sub-optimal input-related demands in terms of transmission, listener and contextual factors would facilitate strategies for improving listening for children with HI above and beyond improvements that could enhance listening for children with typical abilities.

Models of language understanding under challenging conditions, such as the Ease of Language Understanding Model (ELU, Rönnberg et al., 2013), have been successfully used to explain the role of individual differences in cognitive skills related to successful listening. The FUEL also acknowledges the role of cognition in listening and adds the dimension of motivation, providing a mechanism for understanding why some children may simply give up when listening conditions become too challenging. The importance of pleasantness to listening has seldom been recognized (Matthen, 2016) and our findings highlight its possible relevance for the allocation of attention during listening in classrooms.

Teachers should be aware that even low levels of background babble reduce children’s listening comprehension and increase their listening effort, and that hoarseness caused by the teacher raising her voice above background babble may result in the perception of unpleasantness which could, in turn, reduce children’s motivation to keep on making an effort to listen. Individual differences in cognitive function may also explain listening comprehension scores. Teachers should make sure that their face is visible when they speak to children in the classroom but understand that visual cues do not fully compensate the negative effects of the modern interactive classroom.

## Future Work

The findings of the empirical work presented in this perspective article describe how various input-related factors (background babble noise, talker’s voice quality, and the availability of supportive visual cues) influence speech understanding, recall, perceived difficulty and perceived pleasantness by children in conditions similar to what might be found in classroom listening situations. This constellation of findings and the inter-play of factors can be interpreted by applying the FUEL to gain insight into obstacles to optimal listening in activities involved in learning in the classroom, and to inform approaches for solving this multi-faceted problem.

Future work should focus on children who display a range of listener-related factors (including sensory, linguistic and cognitive abilities) to study the effect of motivation on their ability to perform auditory-based tasks imposing varying effort-inducing demands in a simulated classroom setting. Our classroom simulation which manipulates source (voice quality), transmission (multi-talker child babble) and contextual (visual scene) factors provides a promising starting point for future work. In future studies, the intensity and spectral features of source (talker) and transmission (background noise) variables should be evaluated and adjusted to control for potential masking effects caused by spectral overlap of the target voice and competing acoustical signals, while eye-tracking could be used to reveal how visual information is exploited (Sandgren et al., 2015). In addition, transmission factors relating to room acoustics (reverberation) and assistive hearing technologies need to be taken into account, as well as message factors including semantic content. The CELF 4 seems to be a suitable and ecologically valid tool to study the kind of effortful listening activity that arises in the classroom. However, it may be advisable to revise the questions with regard to the cognitive and linguistic skills of the target group as well as their cultural knowledge, and to increase the number of questions to improve psychometric quality (Denman et al., 2017; Nirme et al., 2018).

According to the FUEL, it is likely that relevant activities become prioritized for resource allocation depending on the individual’s motivation. Knowledge is sparse about what motivates children to listen in the classroom. However, pupils’ motivation may be related to the intrinsic (e.g., Johnsrude et al., 2013) and extrinsic (Eckert et al., 2016) value of a voice as well as to the personality traits (Morton and Watson, 2001; Brännström et al., 2015; Matthen, 2016) and emotions (Vaish et al., 2008) attributed to the talker on the basis of voice quality. Based on the results of the present study, we suggest that the unpleasantness of the dysphonic voice may affect children’s motivation to listen to a teacher. Our perspective is that FUEL for children is a useful guide in the continued theoretically driven yet ecologically valid investigation of the challenges involved in learning by looking and listening. As with any framework, future empirical studies could be useful for refining the framework either by supporting or raising questions about the details of how different components of the FUEL operate and interact.

## Author Contributions

BS, VL-Å and JB conceived and designed the empirical project and oversaw data collection. JN developed the virtual talker used in the experiments and analyzed the data. MP-F advised on application of the theoretical model (FUEL) to the group under investigation. MR and BS wrote the first draft of the paper and all authors were actively involved in preparing the final draft.

## Conflict of Interest Statement

The authors declare that the research was conducted in the absence of any commercial or financial relationships that could be construed as a potential conflict of interest.
